# From the heart: hand over heart as an embodiment of honesty

**DOI:** 10.1007/s10339-014-0606-4

**Published:** 2014-03-13

**Authors:** Michal Parzuchowski, Aleksandra Szymkow, Wieslaw Baryla, Bogdan Wojciszke

**Affiliations:** SWPS, University of Social Sciences and Humanities, ul. Polna 16/20, 81-745 Sopot, Poland

**Keywords:** Embodiment, Honesty, Gestures, Hand over heart

## Abstract

Motor movements increase the accessibility of the thought content and processes with which they typically co-occur. In two studies, we demonstrate that putting a hand on one’s heart is associated with honesty, both perceived in others and shown in one’s own behavior. Target persons photographed when performing this gesture appeared more trustworthy than the same targets photographed with both hands down (Study 1). Participants who put their hand on their hearts were more willing to admit their lack of knowledge (Study 2), compared to when they performed a neutral gesture. These findings replicate and extend the notion that bodily experience related to abstract concepts of honesty can influence both perceptions of others, and one’s own actions.

## Introduction

Several studies have demonstrated that body movement (e.g., Mussweiler 2006; Meier et al. 2007), gestures (Chandler and Schwarz 2009), facial muscles contractions (Strack et al. 1988; Parzuchowski and Szymkow-Sudziarska 2008), arm movements (e.g., Förster and Strack 1997; Schnall et al. 2008a, b), and hand configurations (Schubert 2004, Schubert and Koole 2009) influence individuals’ thoughts, feelings, and behaviors (see Barsalou 2008; Niedenthal et al. 2005). A large amount of research implies that not only our bodies and its modalities but also the physical environment and the social context of cognition can be used as an important grounding mechanism (see Barsalou 2010). In the research presented in this paper, we test how a body gesture commonly associated with honesty (hand over heart) influences judgments of the self and others.

According to recent models of embodied cognition, people use their concrete bodily sensations to make sense of abstract concepts and the complexities of social life (Barsalou 2009; Landau et al. 2010). As Barsalou points out (2009), abstract concepts are grounded in specific situations, as people tend to produce broad situational content when asked to describe concepts (Barsalou 2009). Over the course of our lives, we experience many social situations and learn that, for example, telling the truth is associated with a person looking directly into our eyes or that giving somebody a hug expresses one’s friendliness toward that person. Such situated conceptualizations constitute the complex configurations of multimodal components, containing visual, auditory, olfactory, proprioceptive, and interoceptive information, which can be viewed as a perceptual pattern (Barsalou 2009). When a component of a given pattern is evoked by the situation, the remaining components are likely to be activated as well, as they have frequently co-occurred with the perceived component in the past. Thus, once entrenched in memory, situated conceptualizations play an important role in social cognition (Barsalou 2009). By increasing the accessibility of the specific concept, they influence thoughts, feelings and judgments to which the concept is applicable (Barsalou 1999, 2009; Niedenthal et al. 2005). For example, hugging somebody—besides expressing friendliness—is also associated with a pattern of perceptual sensations like the experience of warmth, the smell of the person, and so forth. Thus, when people find themselves in a situation where they experience warmth (e.g., they hold a warm cup or are placed in a warm room), they are more likely to perceive others as friendly and kind (IJzerman and Semin 2009, 2010). Similarly, activating the concept of friendliness leads to experiencing warmth (Szymkow et al. 2013), indicating that thinking about a concept involves simulating the relevant perceptual states (Barsalou 2008). Embodied perspective contends that people represent concepts using the same sensations that co-occur with the activation of such concepts (see Riskind 1984; Chandler and Schwarz 2009).

Bodily induced feelings can influence concept activation even when they are primed unobtrusively, and without awareness of their semantic meaning (see Jostmann et al. 2009; Chandler and Schwarz 2009). For example, Chandler and Schwarz (2009), under the guise of studying the influence of hand movements on text comprehension, asked their participants to extend the middle finger (a hostile gesture), or to extend their index finger (a neutral, control gesture). While making the gesture, the participants were asked to indicate their impressions of an ambiguously described person. Even when none of the participants noticed that they had been performing the gesture, those making the hostility-associated gesture perceived the target person as more hostile than controls. Thus, the mere experience of a bodily sensation may activate associated concepts, which in turn may shape information processing. Such sensations can even influence neuroendocrine levels and subsequent behavioral choices. Brief displays of expansive posture typical for the feelings of power lead to increases in testosterone, decreases in cortisol levels, and a higher tolerance for risk, while assuming a contractive and closed posture typical for powerlessness results in an opposite pattern of changes (Carney et al. 2010).

## Embodiment of honesty

People do not always act honestly although they may pretend to do so (Batson et al. 1999). In particular, people may distort the truth when it brings benefit either to themselves or to others, and yields little harm, especially when there is only a small chance of being caught (Bandura 1991; DePaulo 2004). However, honesty increases when moral standards are made salient, either through self-awareness (Duval and Wicklund 1972) or external moral standards (Batson et al. 1999). For example, Mazar et al. (2008) showed that swearing an oath of allegiance to a code that does not even exist (the MIT Honor Code), or attempting to recall norms that people do not remember (The Ten Commandments), still made people more honest, presumably because this drew attention to one’s internal standards of honesty.

Is there a gesture culturally associated with honesty? The present investigation is based on the idea that a hand-over-heart gesture can prime honesty. Many cultures associate the gesture of placing one’s hand on one’s heart with honesty (not bearing arms, appearing to have genuine intentions, giving a word of honor, and pledging allegiance, Eibl-Eibesfeldt 1996). Since Aristotle (Bakalis 2005), people have believed that the heart is the seat of the human mind, and symbolically, it is still used to refer to the emotional or moral core of a human being. If you are asked to “follow your heart,” this would most likely result in a preference for being more open and emotional—resulting in valuing emotions in the process of decision making and describing oneself as an intuitive thinker (see Fetterman and Robinson 2013). Moreover, many languages (e.g., British English, German, Polish or Russian) have idioms that express honesty through a reference to the gesture of putting one’s hand on one’s heart. For example, people might say “from the heart” (or “with all my heart”) to suggest that their statements are honest. In Poland (where the present studies were conducted), not only is “with hand over heart” (“z ręką na sercu”) an idiomatic expression of honesty used at the end of any dubious statement, but the “hand-over-heart” gesture is also a common emphasis of sincere intentions. As a result of this connection, performing the gesture or using the linguistic expression describing the same action while committing to honest behaviors can and do frequently co-occur.

## Present Research

Performing the hand-over-heart gesture primes a broad sense of honesty. When an individual makes a gesture associated with an abstract concept of honesty, they may be more likely to judge others to be more moral, or they may assume that the other individual will act more honestly. In other words, we suggest that the conceptualization of the social context of honesty can in part be grounded in bodily experience from hand manipulation.

In the present research, we investigated whether observing another person performing the hand-over-heart gesture would result in perceiving the person as more truthful than the same person displaying a control, meaningless gesture. Second, we examined whether performing the same gesture influences the behavior of the performers and makes them more honest (ready to admit that they lack some knowledge) compared to persons performing a control gesture unrelated to honesty.

These hypotheses are based on recent research revealing that the use of hand-over-heart extends beyond being merely an emblematic gesture of convenience and is tightly anchored in the real experiences related to abstract honest behaviors. Parzuchowski and Wojciszke (2014) have demonstrated that an unobtrusive displaying or observing of this gesture increases the accessibility of honesty-related concepts, leading to the stronger use of language associated with honesty (Parzuchowski and Wojciszke 2014; Study 1 and 2). This, in turn, leads to increased perceptions of honesty in others and decreases one’s own cheating (Study 4) and the telling of white lies (Study 3), compared to persons performing neutral gestures (Parzuchowski and Wojciszke 2014). In the present research, we attempt to conceptually replicate and extend this work for other perceptions and behaviors described later.

## Study 1

If people associate the hand-over-heart gesture with telling the truth, they should infer that other persons are more honest when they display the gesture, even if the former do not explicitly think about the meaning of the gesture. To test this hypothesis, we asked participants to judge the credibility of a target person who made some not very credible claims while performing the honesty gesture, or not.

## Methods

### Participants and design

**Fig. 1:**
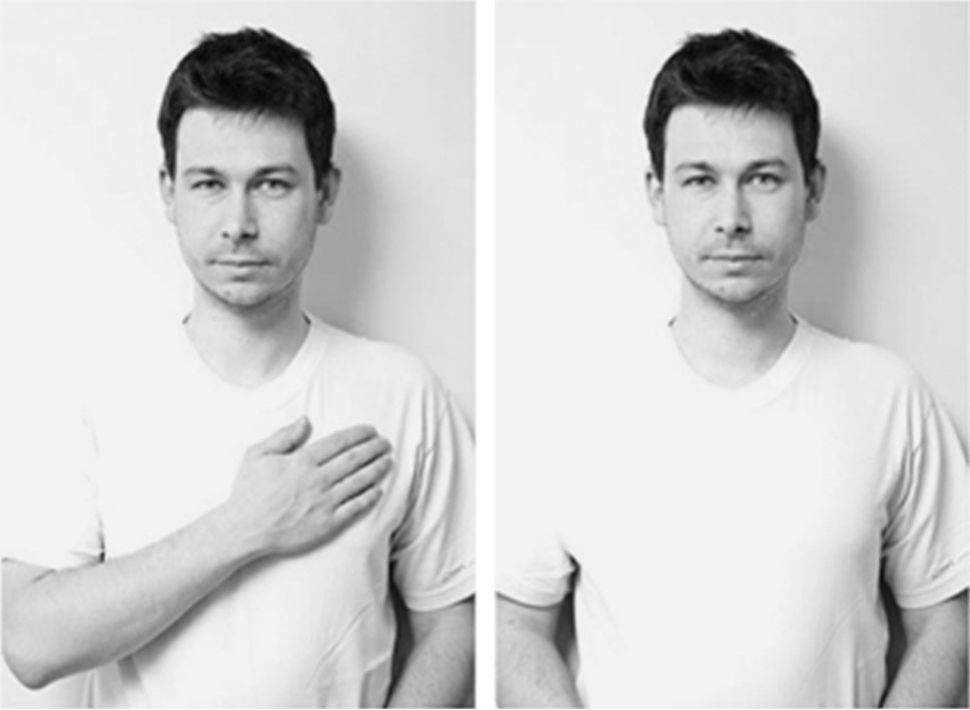
Experiment 1: photographs of target used in Experiment 1

Fifty-five participants (32 female; M_age_ = 25.82; SD = 6.34) completed an online study on person perception. Participants volunteered to participate without monetary reward over a period of 3 days, in response to a study advertisement that was posted on a popular scientific Web site. In this study, they read a paragraph (in Polish) about a young male and accompanying the description was a photograph of the man with either his hand over his heart or both hands down (Fig. 1).^1^

### Procedure and dependent measure

The cover story presented the experiment as a study on the factors influencing impression formation about other’s personality. Participants were asked to read 12 sentences about a young male who was depicted either with or without the gesture of hand over heart. Facial expression, posture, and the target’s lighting were controlled (held identical) between photographs.

The first four sentences presented factual and credible information (i.e., “My name is Piotr and I am 29 years old,” “I am married and I have one child”), followed by eight items taken from the Polish adaptation of the Social Desirability Scale (Drwal and Wilczynska 1995; Crowne and Marlowe 1960) describing socially approved but highly improbable behaviors (“I have never been late to work,” “I never postpone anything to the future,” “I always keep my promises,” “I am kind toward everyone,” “I always respond to letters,” “I have never cheated anyone,” “I have never called in sick,” and “I have never argued with members of my family”*).* Each piece of information was rated on a seven-point scale ranging from 1 (*This is not credible at all*) to 7 (*This is very credible*). This resulted in a reliable index of the target person’s credibility (last eight items: *α* = .93). On the next screen, the participants were asked four other variables about the impression formation^2^ and finally, they were to describe the target’s physical appearance (“What was Piotr’s appearance?” and “Which emotions did Piotr express?”) as well as describe the real purpose of the experiment (“What was the purpose of this study?”), and on the next screen they were thanked for their participation. Then open responses were presented in alphabetical order to a judge who coded them for any mentions of the gesture or a description that matched our hypothesis. Neither of these things were found (no subject mentioned the person’s gesture or described the look of the person in terms relating to the gesture), suggesting that the gesture usage in this context was not remembered and was not associated with the study’s purpose.

## Results and discussion

The analyses focused on the ratings of the 8 improbable social desirability items. As predicted, the participants who saw a young male performing the hand-over-heart gesture rated his credibility as being less dishonest (M = 4.07; SD = 1.56) than those who saw the same target with both hands down (M = 3.25; SD = 1.31), *t*(53) = 2.11, *p* = .039, *d* = .57, 95 % CI (.04, 1.60). This finding supports the notion that merely seeing a target person performing a hand-over-heart gesture increases the target’s credibility. Participants used this gesture as a signal of honesty, and spontaneously incorporated this message into the meaningful impression about the target’s credibility. This pattern of results nicely replicates the previous studies mentioned, as we have previously shown that the hand-over-heart gesture is associated with honesty when participants were either directly asked to interpret the gesture’s meaning (Parzuchowski and Wojciszke 2014; Study 1), or asked to merely observe the photographed person performing this gesture while listening to an audio interview (Parzuchowski and Wojciszke 2014; Study 2). Interestingly, in the current study the usage of the gesture was even less salient, as the photographed person was just a portrayal of the statements’ author, and participants were not asked to pay any particular attention to the photograph.

One reason for this effect might be the similarity of processes underlying perception and action, as assumed by theories of embodied cognition which emphasize that perception partially relies on the perceiver’s own action system (Barsalou 2008; Rizzolatti and Arbib 1998). If so, seeing another person’s gesture produces bodily feedback that could serve as a cue when judging other people’s credibility. In the next study, we examine whether this link influences an individual’s own behavior when they are unobtrusively induced to make the hand-over-heart gesture.

## Study 2

People generally want others to see them in a favorable light (e.g., Goffman 1959; Leary and Kowalski 1990; Sedikides 1993). Consequently, they may be tempted to stretch the truth. For example, people understate their weight in online dating profiles (Toma et al. 2008) and exaggerate their achievements on resumes (Geoghegan 2005), especially when there is little chance of being caught in the lie. People are especially likely to self-enhance when interacting with strangers who have little knowledge of the former’s previous behavior (Tice et al. 1995), or when there is no opportunity to subsequently verify their claims (Schlenker 1975). However, if honesty is brought to mind, people may resist this urge, leading them to make more honest self-presentations. In Study 2, we test whether bodily feedback from an unobtrusive use of hand-over-heart primes honesty by measuring whether people engage in self-enhancing presentations.

## Methods

### Participants and design

Twenty-four right-handed first-year students (22 women; M_age_ = 24.75; SD = 7.29) volunteered to take part in the study in exchange for a course credit. Participants made a hand-over-heart and a control gesture (in a within-subjects-design) while assessing their level of familiarity with some bogus (non-existing) psychological theories.

### Procedure and dependent measure

Participants were told that the study concerned whether people were more likely to remember concepts when learning and recalling information in the same body position. To test this hypothesis, participants were asked to perform two instructed postures while recognizing information that they had previously learned in their psychology classes. In order to unobtrusively manipulate gestures (without priming participants with semantic concepts related to honesty, such as one’s heart), the participants were shown a picture of a body silhouette with four points marked in different colors and letters (ABCD pointing to right hip, right chest, left chest, left hip, respectively) and were asked to stand up and read a note instructing them to place their right hand either on point C (left chest—hand over heart) or D (hand over left hip), while the left hand was always placed on point D. As a result, the participants were standing straight and either placing their right hand over their heart or both of their hands over the left hip. The order of the use of gestures was counterbalanced between the subjects and did not affect the results.

Next, participants learned that recent studies suggested that a large part of our semantic knowledge is acquired implicitly, and therefore, people are not aware that some data (for example, names and authors of psychological theories) are stored in their memory without them intending for it to be so. Since the study concerned this kind of implicit memory, it was important that subjects rate how familiar those theories “felt,” even if they had no explicit knowledge of the theories’ content. This ensured that there was little risk that their claim of knowledge would be challenged, thus increasing the opportunity to self-enhance through an exaggeration of their level of knowledge.

After adopting an instructed posture (hands over left hip or hand over heart), participants watched a slideshow presenting names with respective authors of 11 psychological theories (one at a time for 10 s each) of which the last eight were fictitious (e.g., “Personality integration theory (Hudson 1993),” “Mere fixation theory (Beesly 2002),” “Agentic control theory (Scott and Schrute 2003)”).^3^ Participants were then instructed to judge the familiarity of the presented theories on a seven-point scale ranging from 1 (*I am absolutely unfamiliar with the name*), to 4 (*Hard to say*), to 7 (*I am completely familiar with the name*). Participants’ mood was measured twice (using a short scale from Wojciszke and Baryla 2005), before and after the procedure. Next, participants were thanked and debriefed. Critically, all participants were asked what they thought the study was about and none guessed the correct hypothesis, or mentioned anything about the idea of honesty, or the social meanings of the postures.

## Results and Discussion

We averaged responses of the recognition judgments on the first three (true) theories and the next eight (fictitious) theories and then used repeated measures ANOVA with two factors 2 (gesture used: hand over heart vs. control gesture) × 2 (type of material: true vs. fictitious theory), which yielded an expected main effect of the type of material recognized, *F*(1, 23) = 132.11, *p* = .001, *ηp* ^2^ = .85. This means that participants correctly declared that they more often recognized the true theories (M = 4.86; SD = 1.26) than they did the bogus ones (M = 2.73; SD = 1.12). However, this effect was qualified by an interaction between the type of material and the type of gesture, *F*(1, 23) = 5.37, *p* = .03, *ηp* ^2^ = .19. Participants placing their hands over their hearts claimed that they felt less familiar with the bogus theories (M = 2.50; SD = .96) than they did when placing their hands over their left hip [M = 2.96; SD = 1.29; *t*(23) = 2.08, *p* = .049, *d* = .42, 95 % CI (.001, .93)], while this was not true when participants declared their knowledge of the true theories (M_hand-over-heart_ = 4.90; SD = 1.23; M_control_ = 4,81; SD = 1.30, *t* < 1), suggesting that these differences do not reflect a general tendency to respond in a biased manner. Importantly, the participants’ mood was unaffected by the use of gesture (*t* < 1), suggesting that the accuracy of self-presentation was not driven by changes in the participants’ mood.

In sum, Study 2 suggests that placing one’s hand over one’s heart decreased the students’ self-enhancement behavior when stating their knowledge of bogus psychological theories. The hand-over-heart gesture makes people less willing to over-claim their knowledge, therefore, more honest. This result is complementary to recent findings that other embodied cues can make people less moral. For example, assuming (consciously or inadvertently) an expansive pose typical for power increases stealing, cheating, and traffic violations, and power posing influences these dishonest behaviors through an increased subjective sense of power (Yap et al. 2013).

Interestingly, the results of Study 2 conceptually replicate the previous findings that deception is decreased through the unobtrusive use of the hand-over-heart gesture (Parzuchowski and Wojciszke 2014; Study 3 and 4), while it also expands the drawn conclusions because this behavior was clearly not limited to honesty toward others—participants displayed the self-motivated honesty when claiming one’s own knowledge (they were not externally motivated to limit their self-enhancement). Study 2 also expands the previous findings as it shows that this effect is rather short-lasting because the manipulation was successfully implemented within participants.

## General Discussion

Taken together, present findings link bodily feedback and sincerity, demonstrating that people’s level of honesty can be manipulated through the unobtrusive performance of the hand-over-heart gesture. Persons photographed while making the hand-over-heart gesture appeared less dishonest than the same persons performing a control gesture (Study 1). Furthermore, an unobtrusive performance of this gesture leads people to behave more honestly when admitting their ignorance (Study 2). These effects are not mediated by changes in mood (Study 2). Although the results were consistent with our hypotheses, several alternative explanations are possible. One might suggest that the mechanism for obtained results is due to the interceptive feedback provided by manual haptics, which result not only in specific proprioceptive feedback but also in an additional unique cardiac loop, one which involves simply feeling the beat of one’s own heart (Gu et al. 2013). It is unlikely, but possible, that the present effects are not mediated by any bodily change, but by the increase in interoceptive cardiac sensitivity. If feeling one’s own heart beat could have induced an increased fear of being caught lying, then lying, in turn, could have caused increased heart beat frequency and thus elicited a fear of lying, resulting in a haptic-feedback loop that then lead to increased honesty. However, at least three arguments make this explanation of the present findings highly unlikely. First, it is not easy to feel one’s heartbeat through a shirt or a coat when one is not instructed to do so. Second, honesty was also linked with the gesture even when cardiac sensitivity was not possible, because the gesture was only simulated (Study 1). Third—and most importantly—the opportunity of lying in Study 2 was designed to be undetectable (we informed the participants that we would not actually test their level of knowledge), so the fear of getting caught was minimized. Nevertheless, more in-depth examination of this alternative mechanism for the present results could be a promising goal of future research.

Another limitation of the current results is that in Study 1 we presented participants with a situation where there was no reason to suspect the general credibility of the target person, and the statements were not directly related to the person’s trustworthiness. What seems worth addressing in future studies is that participants should judge the target’s credibility (when performing the honesty gesture or not) directly, while also under the context of limited trust (e.g., presenting the targets as students from a competing university).

High-level social concepts, such as morality or honesty, are challenging examples for grounded cognition theories, as they seem to be non-perceptual. Yet, the present studies provide a demonstration that some cultural links match honesty, in an embodied sense, with the usage of a simple gesture. We examined here the role that a gesture plays in both increasing the perceived credibility of others, and in reducing one’s own dishonesty (inflated self—presentation). The present studies highlight that bodily cues can prime related moral constructs without conscious control, both replicating and extending our previous results (Parzuchowski and Wojciszke 2014).

Traditionally, moral judgment was described as a process involving conscious thought and which heavily relied on language and semantic reasoning (Kohlberg 1973). Our studies build on the social intuitionist model (Haidt 2001), which assumes that moral judgment involves instant intuitions, which are automatic and amenable to contextual cues that can change moral judgment without intention or awareness (cf. Schnall et al. 2008a, b). However, present studies also extend the social intuitionist model, which presumes that moral intuitions are always affective in nature and necessarily involve changes in the affective states of the moral “judge.” We showed that the hand-over-heart gesture can change moral judgment without influencing emotional states (at least mood), because of the mere association between the gesture and certain moral concepts (as evidenced by Parzuchowski and Wojciszke 2014). In our reading, this suggests that moral intuitions are not necessarily affective in nature—rather they are based on associative architecture which is typical for the automatic/impulsive processes (including affective responses), as opposed to controlled/reflective processes that are based on propositions (Strack and Deutsch 2004) and underlie deliberative moral reasoning. In the last two decades, moral judgment and behavior have become thriving areas of empirical research in social psychology. Curiously, these two topics have rarely been studied under the same theoretical auspices or as parts of the same empirical program, and they now appear to be separate fields (see Haidt 2012; Mikulincer and Shaver 2012). The present line of research studied exactly the same embodied phenomenon (the hand-over-heart gesture and its associations) as an antecedent of both social perception (judging the moral character of others) and behavior (honestly admitting one’s own lack of knowledge). Clearly, the embodiment approach may be a platform, which allows the integration of research on moral judgment and behavior.

Our results have revealed yet another two implications. First, by extending the work by Lakoff and Johnson (1980) in the domain of metaphor comprehension, it seems plausible that an abstract concept of honesty is grounded on a very concrete level and can be primed with an unobtrusive use of bodily feedback from a hand configuration. Second, our results show that bodily feedback is used whenever there is a temptation to behave dishonestly, yet it is not taken into consideration when there is no need to lie. It is worth noting, however, that the theory of situated conceptualizations (Barsalou 2009) emphasizes the role of social context in the process of associating bodily states with a specific concept (IJzerman and Koole 2011). For example, sitting on a chair in the living room constitutes a very different perceptual pattern (i.e., feeling relaxed) than sitting on a chair at an office desk (i.e., being focused; Barsalou 2009). A multimodal configuration can bring different interpretations depending on the social context in which they appear. Certainly, putting a hand over one’s heart can be a signal for many practical reasons besides truthfulness, when presented in other contexts, such as checking if one’s wallet is in place in a crowded bus. Thus, the results of our studies should not suggest that the hand-over-heart gesture can be exploited in all contexts and situations as a prime for honesty, nor that it is specific only to the concept of honesty. Instead, we are suggesting that in line with the embodiment perspective (Barsalou 1999), these findings imply that the modal perceptual symbols that compose our knowledge of the concept of honesty involve, among other things, a pattern of specific muscle activation that is used to signal sincere intentions with a hand-over-heart gesture.

Numerous studies document that bodily states can affect a participant’s behavior through the alteration of their emotional states. For example, approach-oriented behaviors, such as when participants pull objects toward themselves or they nod their heads, increase the participants’ positive inclination for the objects or persuasive messages (Wegner et al. 1994; Chen and Bargh 1999; Briñol and Petty 2003). When participants hunch (as oppose to standing upright), they declare more negative feelings (Riskind and Gotay 1982). Unobtrusive contraction of the “smile muscles” increases the declared amusement of the studied material (Strack et al. 1988), and the head tilting upward induces a feeling of pride (Stepper and Strack 1993). What is new about our research is that our results (Study 2) indicate that bodily movements can also affect the social behavior of honest self-presentation without altering the affective state.

This line of research enhances social embodied cognition, as it demonstrates how gestures can not only enhance the comprehension of spontaneous language production (Morford and Goldin-Meadow 1992), but simultaneously alter the speaker’s behavior. Our results are important, as they demonstrate how embodied theories can accommodate for findings obtained in socially based situations, relating to the way people perceive and express honesty. In sum, the bodily experience of abstract moral metaphors can not only influence the actors’ perception of their social environment but also the actors’ own actions.

## Footnotes

1 In line with Simmons et al. (2012) prescription, we report how we determined our sample size, all data exclusions (if any), all manipulations, and all measures in the study.

2 After the credibility judgments were collected, participants were asked to judge the target’s level of agency (12 items) and communion (12 items) traits, and willingness to involve the target into agentic (three items) and communal (three items) activities. Those measures were not affected by the manipulation (*t* < 1), which might suggest the specificity of the credibility rating, but there might be an even simpler interpretation of that pattern: following measures were time consuming, and because the picture was displayed only on the first screen, the gesture manipulation simply faded away with time. Because of that confound, these DV’s are not discussed in detail.

3 Participants saw the three true theories first (the first theory contained the true author and the correct (famous) theory name, while the next two theories contained only the real name of a well-known author, but the name of the theory was slightly altered), as we wanted to make the fake ones that were presented subsequently to seem more plausible.

## References

[CR1] Bakalis A (2005). Handbook of Greek philosophy: from Thales to the Stoics analysis and fragments.

[CR2] Bandura A, Kurtines WM, Gewirtz JL (1991). Social cognitive theory of moral thought and action. Handbook of moral behavior and development.

[CR3] Barsalou LW (1999). Perceptual symbol systems. Behav Brain Sci.

[CR4] Barsalou LW (2008). Grounded cognition. Annu Rev Psychol.

[CR5] Barsalou LW, Robbins P, Aydede M (2009). Situating concepts. Cambridge handbook of situated cognition.

[CR6] Barsalou LW (2010). Grounded cognition: past, present, and future. Topics Cogn Sci.

[CR7] Batson CD, Thompson ER, Seuferling G, Whitney H, Strongman JA (1999). Moral hypocrisy: appearing moral to oneself without being so. J Pers Soc Psychol.

[CR02] Beesly P (2002) Mere fixation theory. Personal Bull Manag Stud 3:102–106

[CR8] Briñol P, Petty RE (2003). Overt head movements and persuasion: a self-validation analysis. J Pers Soc Psychol.

[CR9] Carney DR, Cuddy AJ, Yap AJ (2010). Power posing brief nonverbal displays affect neuroendocrine levels and risk tolerance. Psychol Sci.

[CR10] Chandler J, Schwarz N (2009). How extending your middle finger affects your perception of others: learned movements influence concept accessibility. J Exp Soc Psychol.

[CR11] Chen M, Bargh JA (1999). Consequences of automatic evaluation: immediate behavioral predispositions to approach or avoid the stimulus. Pers Soc Psychol Bull.

[CR12] Crowne DP, Marlowe D (1960). A new scale of social desirability independent of psychopathology. J Consult Psychol.

[CR13] DePaulo BM, Miller AG (2004). The many faces of lies. The social psychology of good and evil: understanding our capacity for kindness and cruelty.

[CR14] Drwal RL, Wilczynska J, Brzozowski P, Oles P (1995). Opracowanie Kwestionariusza Aprobaty Społecznej (KAS) [Development of Social Acceptance Questionnaire]. Adaptacja kwestionariuszy osobowości.

[CR15] Duval TS, Wicklund R (1972). A theory of objective self-awareness.

[CR16] Eibl-Eibesfeldt I (1996). Love and hate. The natural history of behavior patterns.

[CR17] Fetterman AK, Robinson MD (2013). Do you use your head or follow your heart? Self-location predicts personality, emotion, decision making, and performance. J Pers Soc Psychol.

[CR18] Förster J, Strack F (1997). Motor actions in retrieval of valenced information: a motor congruency effect. Percept Mot Skills.

[CR19] Geoghegan T (2005) The CV detectives. BBC News Mag. Retrieved from http://news.bbc.co.uk/2/hi/uk_news/magazine/4167204.stm

[CR20] Goffman E (1959). The presentation of self in everyday life.

[CR21] Gu J, Zhong CB, Page-Gould E (2013). Listen to your heart: when false somatic feedback shapes moral behavior. J Exp Psychol Gen.

[CR22] Haidt J (2001). The emotional dog and its rational tail: a social intuitionist approach to moral judgment. Psychol Rev.

[CR23] Haidt J (2012). The righteous mind. Why good people are divided by politics and religion.

[CR01] Hudson S (1993) Personality integration among obese individuals. Health Issues 5:10–15

[CR24] IJzerman H, Koole SL (2011). From perceptual rags to metaphoric riches—bodily, social, and cultural constraints on sociocognitive metaphors: comment on Landau, Meier, and Keefer (2010). Psychol Bull.

[CR25] IJzerman H, Semin GR (2009) The thermometer of social relations: mapping social proximity on temperature. Psychol Sci 10:1214–1220 10.1111/j.1467-9280.2009.02434.x 19732385

[CR26] IJzerman H, Semin GR (2010) Temperature perceptions as a ground for social proximity. J Exp Soc Psychol 46:867–873

[CR27] Jostmann NJ, Lakens D, Schubert TW (2009). Weight as an embodiment of importance. Psychol Sci.

[CR28] Kohlberg L (1973). The claim to moral adequacy of a highest stage of moral judgment. J Philos.

[CR29] Lakoff G, Johnson M (1980). Metaphors we live by.

[CR30] Landau MJ, Meier BP, Keefer LA (2010). A metaphor-enriched social cognition. Psychol Bull.

[CR31] Leary MR, Kowalski RM (1990). Impression management: a literature review and two-component model. Psychol Bull.

[CR32] Mazar N, Amir O, Ariely D (2008). The dishonesty of honest people: a theory of self-concept maintenance. J Mark Res.

[CR33] Meier BP, Hauser DJ, Robinson MD, Friesen CK, Schjeldahl K (2007). What’s “up” with God? Vertical space as a representation of the divine. J Pers Soc Psychol.

[CR34] Mikulincer M, Shaver PR (2012). The social psychology of morality.

[CR35] Morford M, Goldin-Meadow S (1992). Comprehension and production of gesture in combination with speech in one-word speakers. J Child Lang.

[CR36] Mussweiler T (2006). Doing is for thinking! Stereotype activation by stereotypic movements. Psychol Sci.

[CR37] Niedenthal PM, Barsalou L, Winkielman P, Krauth-Gruber S, Ric F (2005). Embodiment in attitudes, social perception, and emotion. Pers Soc Psychol Rev.

[CR38] Parzuchowski M, Szymkow-Sudziarska A (2008). Well, slap my thigh: expression of surprise facilitates memory of surprising material. Emotion.

[CR39] Parzuchowski M, Wojciszke B (2014). Hand over heart primes moral judgments and behavior. J Nonverbal Behav.

[CR40] Riskind JH (1984). They stoop to conquer: guiding and self-regulatory functions of physical posture after success and failure. J Pers Soc Psychol.

[CR41] Riskind JH, Gotay CC (1982). Physical posture: could it have regulatory or feedback effects on motivation and emotion?. Motiv Emot.

[CR42] Rizzolatti G, Arbib MA (1998). Language within our grasp. Trends Neurosci.

[CR43] Schlenker BR (1975). Self-presentation: managing the impression of consistency when reality interferes with self-enhancement. J Pers Soc Psychol.

[CR44] Schnall S, Haidt J, Clore GL, Jordan AH (2008). Disgust as embodied moral judgment. Pers Soc Psychol Bull.

[CR45] Schnall S, Benton J, Harvey S (2008). With a clean conscience: cleanliness reduces the severity of moral judgments. Psychol Sci.

[CR46] Schubert T (2004). The power in your hand: gender differences in bodily feedback from making a fist. Pers Soc Psychol Bull.

[CR47] Schubert TW, Koole SL (2009). The embodied self: making a fist enhances men’s power-related self-conceptions. J Exp Soc Psychol.

[CR03] Scott M, Schrute D (2003) Agentic control over intrinsic thoughts. Pap Stud Psychol 7:56–69

[CR48] Sedikides C (1993). Assessment, enhancement, and verification determinants of the self-evaluation process. J Pers Soc Psychol.

[CR49] Simmons JP, Nelson LD, Simonsohn U (2012) A 21 word solution. http://ssrn.com/abstract=2160588

[CR50] Stepper S, Strack F (1993). Proprioceptive determinants of emotional and nonemotional feelings. J Pers Soc Psychol.

[CR51] Strack F, Deutsch R (2004). Reflective and impulsive determinants of social behavior. Pers Soc Psychol Rev.

[CR52] Strack F, Martin LL, Stepper S (1988). Inhibiting and facilitating condition of facial expressions: a non-obtrusive test of the facial feedback hypothesis. J Pers Soc Psychol.

[CR53] Szymkow A, Chandler J, Ijzerman H, Parzuchowski M, Wojciszke B (2013). Warmer hearts, warmer rooms: focusing on positive communal but not agentic traits increases estimates of ambient temperature. Soc Psychol.

[CR54] Tice DM, Butler JL, Muraven M, Stillwell AM (1995). When modesty prevails: differential favorability of self-presentation to friends and strangers. J Pers Soc Psychol.

[CR55] Toma C, Hancock J, Ellison N (2008). Separating fact from fiction: an examination of deceptive self-presentation in online dating profiles. Pers Soc Psychol Bull.

[CR56] Wegner DM, Lane JD, Dimitri S (1994). The allure of secret relationships. J Pers Soc Psychol.

[CR57] Wojciszke B, Baryla W (2005). Skale do pomiaru nastroju i szesciu emocji [Scales for the measurement of mood and six emotions]. Czasopismo Psychologiczne.

[CR58] Yap AJ, Wazlawek AS, Lucas BJ, Cuddy AJC, Carney DR (2013). The ergonomics of dishonesty : the effect of incidental posture on stealing, cheating, and traffic violations. Psychol Sci.

